# Cathepsin L induces cellular senescence by upregulating CUX1 and p16^INK4a^

**DOI:** 10.18632/aging.205955

**Published:** 2024-06-18

**Authors:** Yuwei Wu, Danli Jiang, Qing Liu, Shaoyang Yan, Xiuzhen Liu, Ting Wu, Wei Sun, Gang Li

**Affiliations:** 1 Department of Cardiology, Third Xiangya Hospital, Central South University, Changsha, China; 2 Aging Institute, University of Pittsburgh, Pittsburgh, PA 15260, USA; 3 International Center for Aging and Cancer Hainan Medical University, Hainan, China; 4 Tsinghua Medicine, Tsinghua University, Peking, China; 5 Department of Medicine, Division of Cardiology, University of Pittsburgh School of Medicine, Pittsburgh, PA 15213, USA; 6 Vascular Medicine Institute, University of Pittsburgh School of Medicine and University of Pittsburgh Medical Center, Pittsburgh, PA 15213, USA

**Keywords:** senescence, atherosclerosis, p16^INK4a^, Cathepsin L, CUX1

## Abstract

Cathepsin L (CTSL) has been implicated in aging and age-related diseases, such as cardiovascular diseases, specifically atherosclerosis. However, the underlying mechanism(s) is not well documented. Recently, we demonstrated a role of CUT-like homeobox 1 (CUX1) in regulating the p16^INK4a^-dependent cellular senescence in human endothelial cells (ECs) and vascular smooth muscle cells (VSMCs) via its binding to an atherosclerosis-associated functional SNP (fSNP) rs1537371 on the *CDKN2A/B* locus. In this study, to determine if CTSL, which was reported to proteolytically activate CUX1, regulates cellular senescence via CUX1, we measured the expression of CTSL, together with CUX1 and p16^INK4a^, in human ECs and VSMCs undergoing senescence. We discovered that CUX1 is not a substrate that is cleaved by CTSL. Instead, CTSL is an upstream regulator that activates *CUX1* transcription indirectly in a process that requires the proteolytic activity of CTSL. Our findings suggest that there is a transcription factor in between CTSL and CUX1, and cleavage of this factor by CTSL can activate CUX1 transcription, inducing endothelial senescence. Thus, our findings provide new insights into the signal transduction pathway that leads to atherosclerosis-associated cellular senescence.

## INTRODUCTION

Cathepsin L (CTSL) is a lysosomal acid cysteine protease primarily responsible for the breakdown of proteins [1]. It is expressed in different types of cells in all tissues and is crucial in diverse biological processes including autophagy, antigen presentation, proprotein activation, and apoptosis [2]. Besides being involved in cancer development [3], CTSL was also implicated in other diseases such as chronic kidney disease [4], cardiovascular diseases [5, 6], and type 2 diabetes [7]. Particularly, studies from CTSL-deficient mice concluded that CTSL plays a direct role in atherosclerosis [8]. All these abovementioned diseases are considered as age-related diseases as the incidence of these diseases rises exponentially along with age; therefore, aging is considered as the single risk factor for all these diseases. Among several hallmarks that contribute to aging, accumulation of senescent cells is believed to be one of the major culprits of aging and age-related diseases [9]. Together, this suggests that CTSL may be involved in the pathogenesis of age-related diseases by regulating cellular senescence. However, so far, few reports investigated the role of CTSL in regulating cellular senescence.

CUT-like homeobox 1 (CUX1), as a homeodomain transcription factor, participates in tissue development, cell migration, proliferation, differentiation, and DNA damage repair [10, 11]. Based on post-genome-wide association studies (GWAS) functional studies on the aging-related *CDKN2A/B* locus [12], we recently demonstrated that CUX1 is an activator of p16^INK4a^, regulating p16^INK4a^-dependent cellular senescence via its binding to an atherosclerosis-associated functional SNP (fSNP) rs1537371 on the *CDKN2A/B* locus [13]. Interestingly, it was previously reported that CUX1 can be proteolytically activated by CTSL [10, 14, 15], which generates a truncated, but transcriptionally more active CUX1 isoform with an apparent molecular weight of 110 kDa, instead of 200 kDa for the full length CUX1. Together, these data suggest that CTSL may regulate cellular senescence via proteolytically activating CUX1.

Based on the initiating trigger, cellular senescence can be classified as replicative or stress-induced senescence. The former is induced by telomeric shortening occurring with each round of cell division when telomeric shortening exceeds a critical threshold [16–18]. Stress-induced senescence, also known as premature senescence, can be triggered by a variety of factors, including ionizing irradiation, chemotherapeutic reagents, oxidative stress stimulated by exogenous reactive oxygen species (ROS) exposure, oncogene activation, or oxidized low-density lipoprotein (ox-LDL) [19–21]. Both replicative and stress-induced senescence are mediated through p16^INK4a^/RB (retinoblastoma protein) pathway [22–25], however, the signaling pathway that leads to the p16^INK4a^-dependent cellular senescence is largely unknown.

In this present study, to determine if CTSL indeed plays a role in regulating cellular senescence, possibly by proteolytically activating CUX1 as previously reported [10, 14, 15], we investigated the expression of CTSL, CUX1, and p16^INK4a^ in both early and late passage human endothelial cells (ECs) and vascular smooth muscle cells (VSMCs). We discovered that CTSL and CUX1 as well as p16^INK4a^ are all upregulated at both mRNA and protein levels in late versus early passage human ECs and VSMCs, indicating that CUX1 is not an enzymatic substrate of CTSL. Instead, we demonstrated that CTSL is an upstream regulator of CUX1, activating CUX1 transcription indirectly, and, therefore, regulating cellular senescence. While our findings provide a better understanding of the signal transduction pathway of cellular senescence, the mechanism underlying the induction of CTSL and the regulation of CUX1 are discussed.

## RESULTS

### CUX1 is not a substrate that is cleaved by CTSL in human ECs and VSMCs

**Figure 1:**
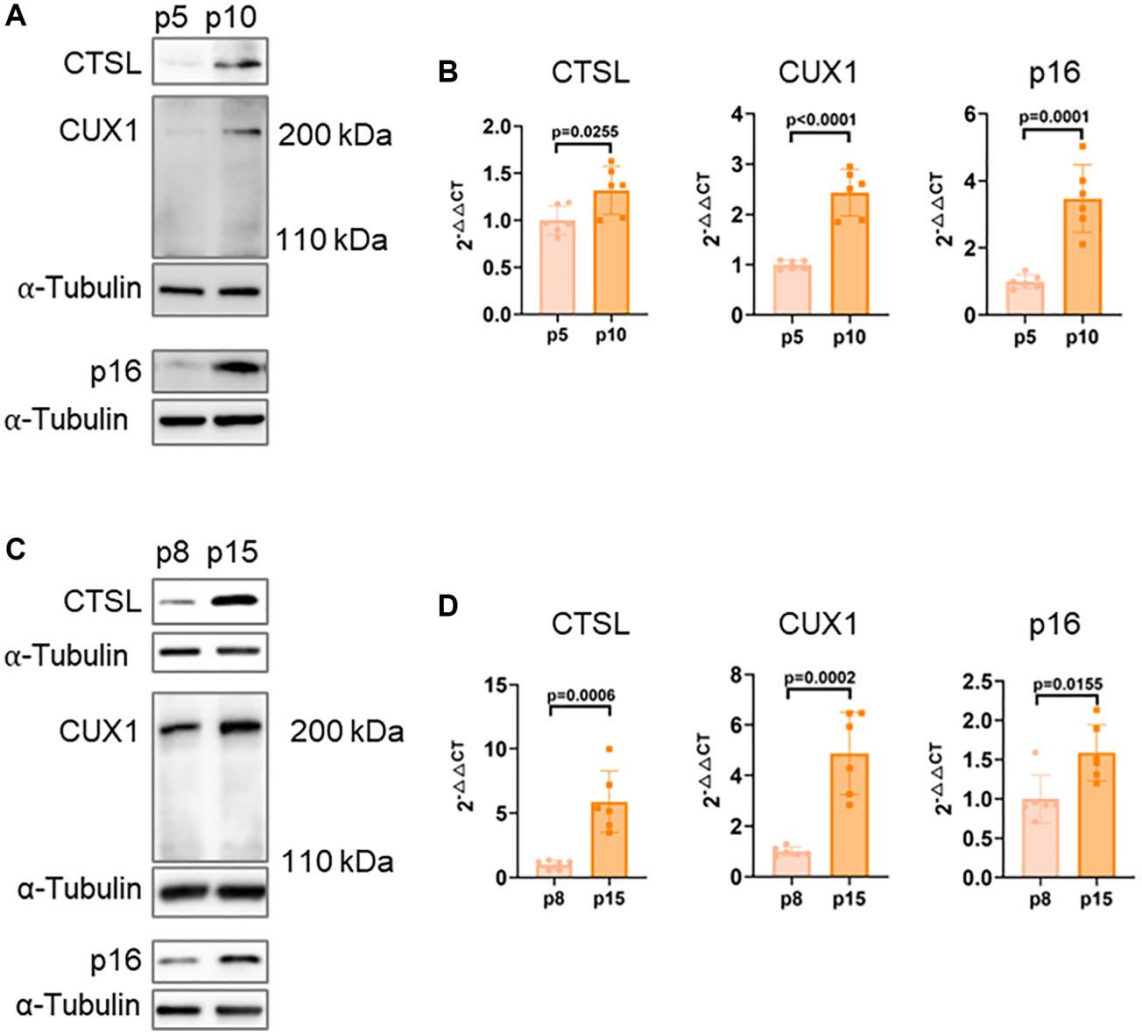
**Correlated expression of CTSL, CUX1 and p16^INK4a^ in human ECs and VSMCs.** (**A**, **B**) Western blots and qPCR analysis showing upregulation of CTSL, CUX1 and p16^INK4a^ in late passage (p10) versus early passage (p5) human ECs. (**C**, **D**) Western blots and qPCR analysis confirming upregulation of CTSL, CUX1 and p16^INK4a^ in late passage (p15) versus early passage (p8) human VSMCs. Data for Western blots represent three biologically independent samples (*n* = 3). Data for qPCR represent a combination of three (*n* = 3) biologically independent experiments.

Recently, our post-GWAS functional studies demonstrated that CUX1 is an activator of p16^INK4a^, regulating p16^INK4a^-dependent cellular senescence, and, consistently, an increased expression of the full length CUX1 in late passage (p10) human ECs was observed compared to early passage (p5) human ECs [13]. Since previous reports showed that CTSL can proteolytically cleave CUX1 to produce a truncated, but more active form of CUX1 with an apparent molecular weight of 110 kDa, instead of 200 kDa for the full length CUX1, in human cancer cells [10, 14, 15], we therefore hypothesized that CTSL expression should be lower in p10 human ECs than that in p5 human ECs. To test this, we first investigated the expression of CTSL as well as CUX1 and p16^INK4a^ in p5 and p10 human ECs. To our surprise, an increased expression of CTSL as well as CUX1 and p16^INK4a^ in p10 versus p5 human ECs was detected by both Western blots and qPCR analyses and no truncated CUX1 at 110 kDa was detected (Figure 1A, 1B). The same analyses were also performed in human VSMCs and similar results were observed, demonstrating that an increased expression of CTSL, CUX1 and p16^INK4a^ in late passage (p15) versus early passage (p8) human VSMCs as shown in Figure 1C, 1D. Together, these data demonstrate that CUX1 is not a substrate of CTSL, at least in human ECs and VSMCs, as both CTSL and CUX1 expression are passage-dependent with an increased expression in late versus early passage human ECs and VSMCs. The same data also demonstrate that CTSL as well as CUX1 and p16^INK4a^ are all activated by transcription as activation can be detected by qPCR analysis.

### CTSL is an upstream activator of CUX1, regulating cellular senescence

**Figure 2:**
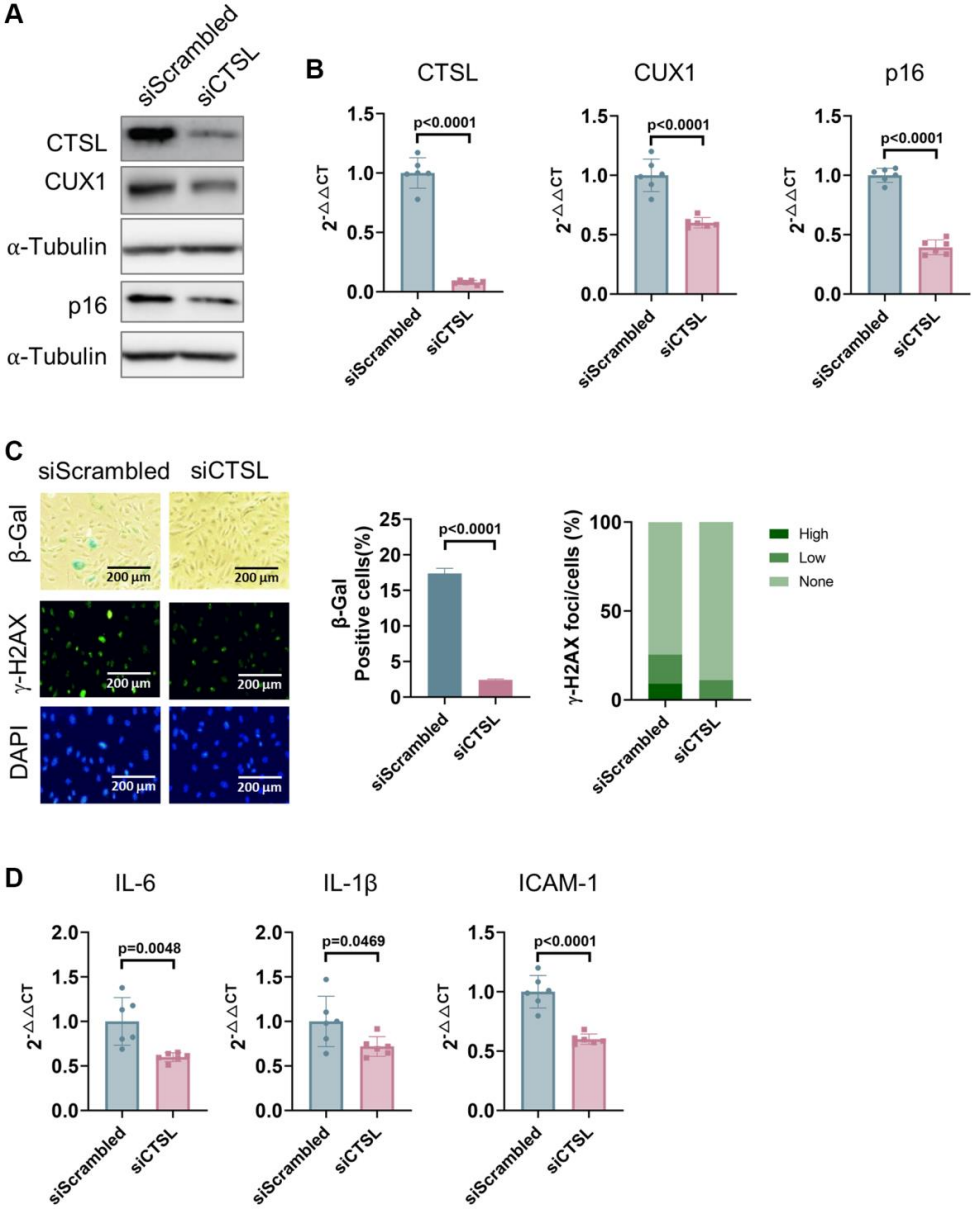
**Downregulation of CTSL decreases the expression of CUX1 and p16^INK4a^, and inhibits cellular senescence in human ECs.** (**A**, **B**) Western blot and qPCR analyses showing that siRNA-mediated CTSL knockdown (siCTSL) downregulates CUX1 and p16^INK4a^ expression in human ECs. (**C**, **D**) siRNA-mediated CTSL knockdown (siCTSL) suppresses cellular senescence in human ECs as evidenced by SA-β-gal and γ-H2AX staining as well as by the expression of the SASP genes *IL6*, *IL-1β*, and *ICAM-1*. Quantitative plots for both β-gal^+^ cells (%) in SA-β-gal staining and γ-H2AX foci/cells (%) with γ-H2AX staining were shown on the right side of the panel C. DAPI staining visualizes the presence of nuclei as a control for the cells in this analysis. Data for Western blots represent three biologically independent samples (*n* = 3). Data for qPCR represent a combination of three (*n* = 3) biologically independent experiments. Data for both SA-β-gal staining and γ-H2AX staining represent three biologically independent samples (*n* = 3).

Correlation of expression among CTSL, CUX1, and p16^INK4a^ in late passage human cells suggests a possible functional relevance among these genes. To determine if CTSL is an upstream regulator of CUX1 and p16^INK4a^, we performed siRNA-mediated knockdown of CTSL in p8 human ECs. We detected a significant downregulation of both CUX1 and p16^INK4a^ expression at both protein and mRNA levels in the siRNA-mediated CTSL knockdown ECs as shown in Figure 2A, 2B. These data further demonstrate that CUX1 is not a substrate of CTSL protease activity as, otherwise, siRNA-mediated CTSL knockdown should result in an upregulation of CUX1. The same data also suggest that CUX1 is a downstream effect gene of CTSL as, otherwise, siRNA-mediated CTSL knockdown should not lead to altered expression of CUX1. Consistent with the reduced expression of CUX1 and p16^INK4a^, a significant reduction of cellular senescence in the siRNA-mediated CTSL knockdown ECs was observed as measured by decreased SA-β-gal and γ-H2AX staining (Figure 2C) as well as by decreased expression of the senescence-associated secretory phenotype (SASP) genes *IL6, IL-1β*, and *ICAM-1* (Figure 2D). Therefore, our data demonstrate that CTSL is an upstream activator of CUX1 and/or p16^INK4a^, and downregulation of CTSL suppresses CUX1 and/or p16^INK4a^ as well as cellular senescence, presumably via modulating CUX1 and p16^INK4a^ expression.

### CTSL protease activity is required to activate CUX1 and p16^INK4a^ as well as cellular senescence

**Figure 3:**
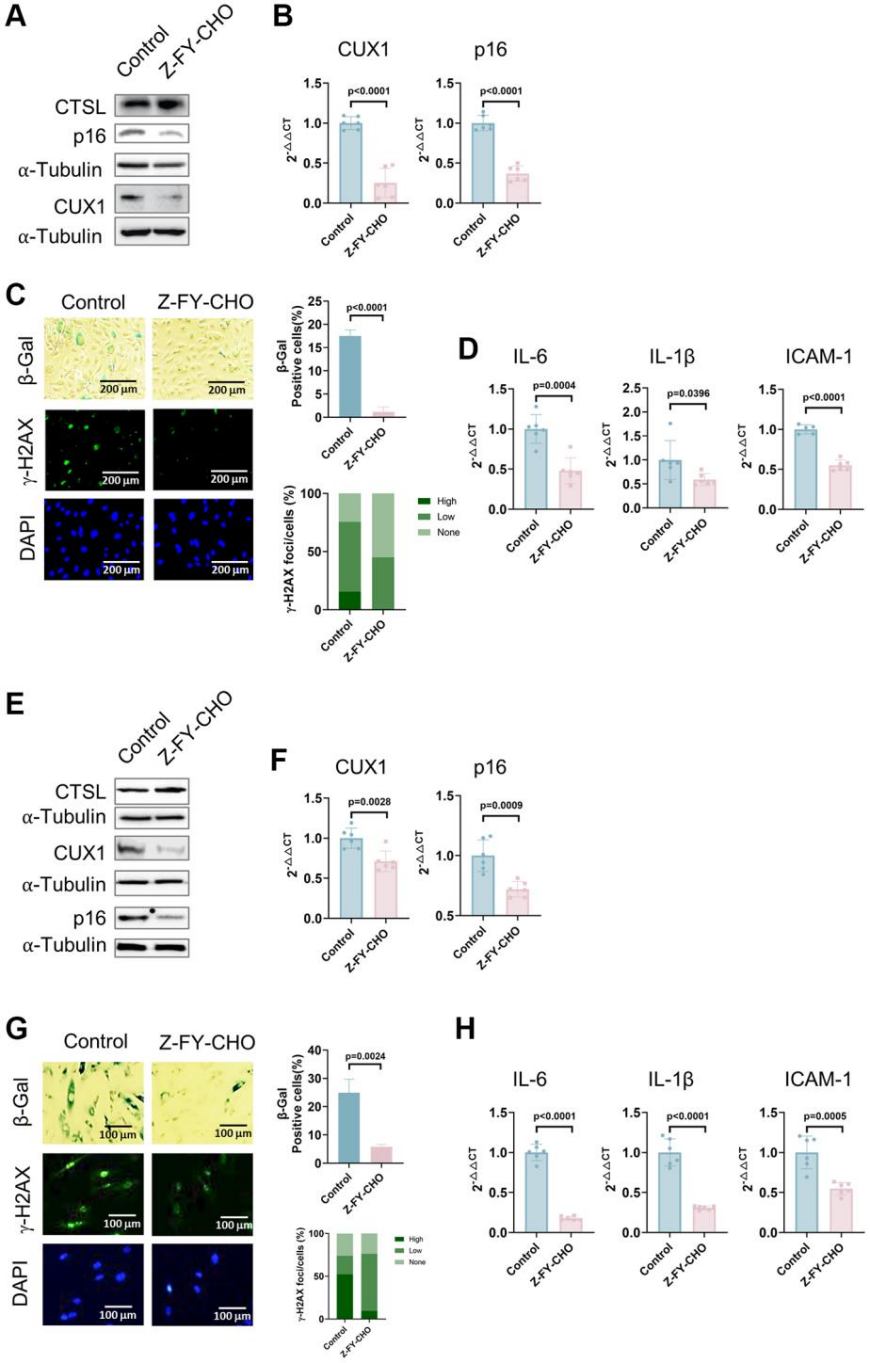
**Inhibition of CTSL protease activity decreases the expression of CUX1 and p16^INK4a^, and inhibits cellular senescence in human ECs and VSMCs.** (**A**, **B**) Western blot and qPCR analyses showing that inhibition of CTSL protease activity by Z-FY-CHO downregulates CUX1 and p16^INK4a^ in human ECs. (**C**, **D**) inhibition of CTSL protease activity by Z-FY-CHO suppresses cellular senescence in human ECs as evidenced by SA-β-gal and γ-H2AX staining as well as by the expression of the SASP genes *IL6*, *IL-1β*, and *ICAM-1*. Quantitative plots for both β-gal^+^ cells (%) in SA-β-gal staining and γ-H2AX foci/cells (%) with γ-H2AX staining were shown on the right side of the panel C. DAPI staining visualizes the presence of nuclei as a control for the cells in this analysis. (**E**, **F**) Western blot and qPCR analyses showing that inhibition of CTSL protease activity by Z-FY-CHO downregulates CUX1 and p16^INK4a^ in human VSMCs. (**G**, **H**) inhibition of CTSL protease activity by Z-FY-CHO suppresses cellular senescence in human VSMCs as evidenced by SA-β-gal and γ-H2AX staining as well as by the expression of the SASP genes *IL6*, *IL-1β*, and *ICAM-1*. Quantitative plots for both β-gal^+^ cells (%) in SA-β-gal staining and γ-H2AX foci/cells (%) with γ-H2AX staining were shown on the right side of the panel G. DAPI staining visualizes the presence of nuclei as a control for the cells in this analysis. Data for Western blots represent three biologically independent samples (*n* = 3). Data for qPCR represent a combination of three (*n* = 3) biologically independent experiments. Data for both SA-β-gal staining and γ-H2AX staining represent three biologically independent samples (*n* = 3).

To further demonstrate that CTSL activates cellular senescence by upregulating CUX1 and/or p16^INK4a^, we also treated p8 human ECs with 30 μM Z-FY-CHO, a potent and selective inhibitor of CTSL enzymatic activity [26]. Consistent with the results observed in the siRNA-mediated CTSL knockdown human ECs (Figure 2A–2D), a significant reduction of both CUX1 and p16^INK4a^ expression at both protein and mRNA levels was observed in the Z-FY-CHO-treated human ECs while, as expected, CTSL expression remained unchanged (Figure 3A, 3B). Consequently, a decreased cellular senescence was observed as measured by the reduced SA-β-gal and γ-H2AX staining (Figure 3C) as well as by the decreased expression of the SASP genes *IL6, IL-1β*, and *ICAM-1* (Figure 3D). The same analyses were also performed in human VSMCs, and we verified that Z-FY-CHO inhibits both CUX1 and p16^INK4a^ expression as well as cellular senescence in human VSMCs as shown in Figure 3E–3H.

Taken together with the data presented in Figures 1 and 2, these data validate that CTSL is an upstream activator of CUX1 and/or p16^INK4a^, and induces cellular senescence, presumably via transcriptionally activating CUX1 and/or p16^INK4a^. At the same time, our findings demonstrate that the proteolytic activity of CTSL is required for the transcriptional activation of CUX1 and/or p16^INK4a^, which suggests that CTSL is not a direct transcriptional factor of CUX1, and CTSL may activate CUX1 transcription by proteolytically activating a currently unknown transcription factor.

### CUX1 is a downstream mediator of CTSL, regulating p16^INK4a^ and p16^INK4a^-dependent cellular senescence

**Figure 4:**
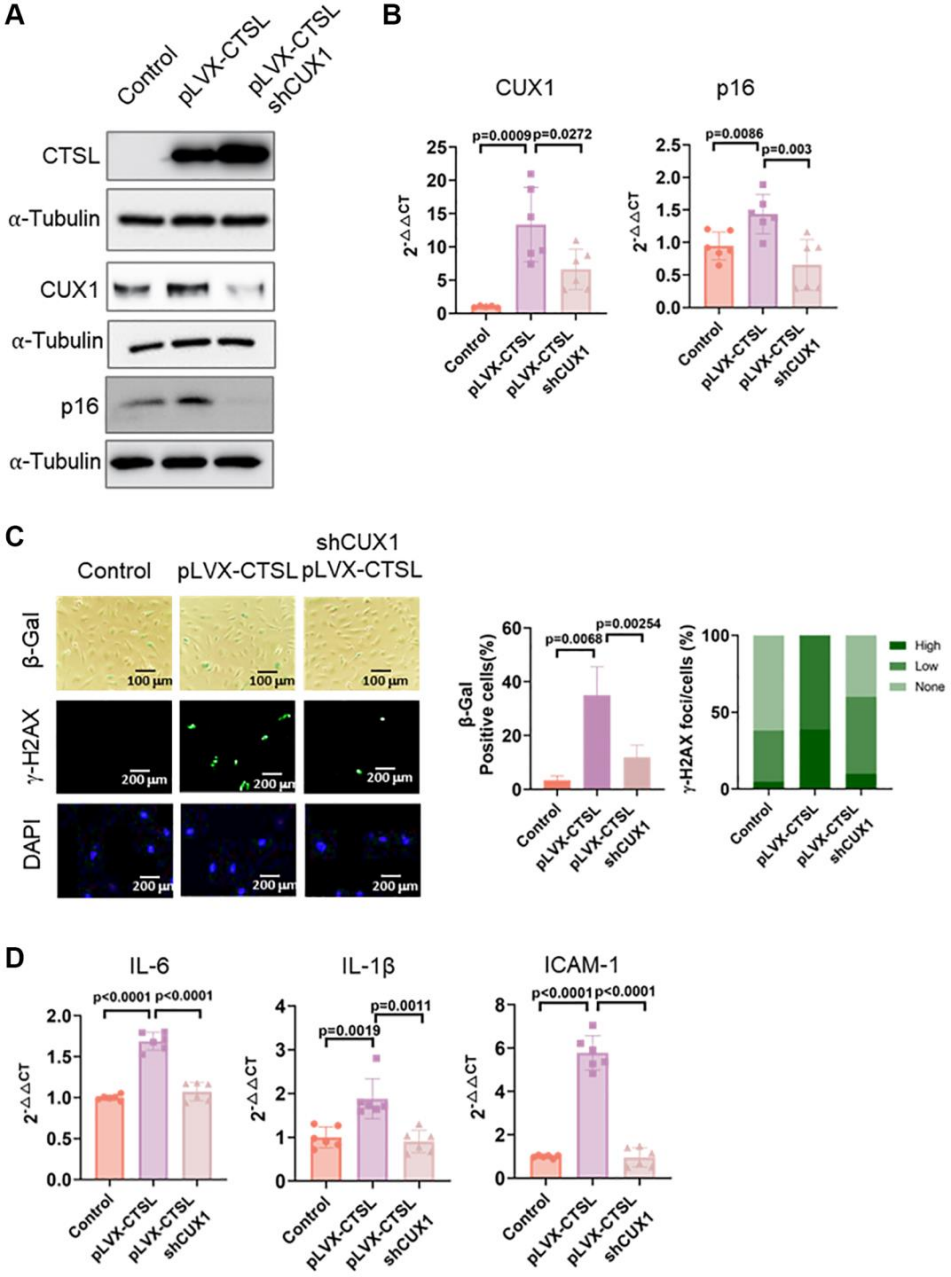
**CTSL is an upstream regulator of CUX1 and p16^INK4a^, inducing replicative senescence.** (**A**, **B**) Western blot and qPCR analyses showing that overexpression of CTSL (pLVX-CTSL) activates CUX1 and p16^INK4a^ expression, and that shRNA-mediated CUX1 knockdown (shCUX1) reverses the increased expression of CUX1 and p16^INK4a^ in CTSL-overexpressing (pLVX-CTSL) human ECs. (**C**, **D**) SA-β-gal and γ-H2AX staining and the expression of SASP genes *IL6*, *IL-1β* and *ICAM-1* verify that overexpression of CTSL (pLVX-CTSL) induces cellular senescence, and that shRNA-mediated CUX1 knockdown (shCUX1) reverses the increased senescence in the CTSL-overexpressing (pLVX-CTSL) human ECs. Quantitative plots for both β-gal^+^ cells (%) in SA-β-gal staining and γ-H2AX foci/cells (%) with γ-H2AX staining were shown on the right side of the panel C. DAPI staining visualizes the presence of nuclei as a control for the cells in this analysis. Data for Western blots represent three biologically independent samples (*n* = 3). Data for qPCR represent a combination of three (*n* = 3) biologically independent experiments. Data for both SA-β-gal staining and γ-H2AX staining represent three biologically independent samples (*n* = 3).

To confirm that CUX1 is the downstream mediator of CTSL, regulating p16^INK4a^ as well as p16^INK4a^-dependent cellular senescence in human ECs, as we previously reported [13], we performed a functional complementation assay. We first overexpressed CTSL in human ECs using a lentiviral expression vector pLVX-CTSL. Overexpression of CTSL was confirmed by Western blot as shown in Figure 4A (Lane 1 and 2). As a result, upregulation of both CUX1 and p16^INK4a^ was validated at both mRNA and protein levels (Figure 4A, 4B, Lane 1 and 2). Consistent with the increased expression of CUX1 and p16^INK4a^, CTSL-overexpressed human ECs showed an increased level of cellular senescence as measured by both the enhanced SA-β-gal and γ-H2AX staining (Figure 4C, Column 1 and 2) as well as by the increased expression of the SASP genes *IL6* and *IL-1β*, and *ICAM-1* (Figure 4D, Lane 1 and 2). Next, we performed shRNA-mediated CUX1 knockdown in the CTSL-overexpressing human ECs as previously described [13]. After viral infection, downregulation of CUX1 was first confirmed by both Western blot and qPCR analyses (Figure 4A, 4B, Lane 2 and 3). As a result, downregulation of p16^INK4a^ was also detected (Figure 4A, 4B, Lane 2 and 3) and, correspondingly, a decreased cellular senescence was observed by the decreased staining of both SA-β-gal and γ-H2AX (Figure 4C, Column 2 and 3) as well as by the decreased expression of the SASP genes *IL6* and *IL-1β*, and *ICAM-1* (Figure 4D, Lane 2 and 3). Thus, these data demonstrate that CUX1 is a downstream mediator of CTSL, regulating p16^INK4a^ and cellular senescence in response to CTSL activation.

### CTSL regulates premature senescence induced by bleomycin and oxidized low density lipoprotein (oxLDL)

**Figure 5:**
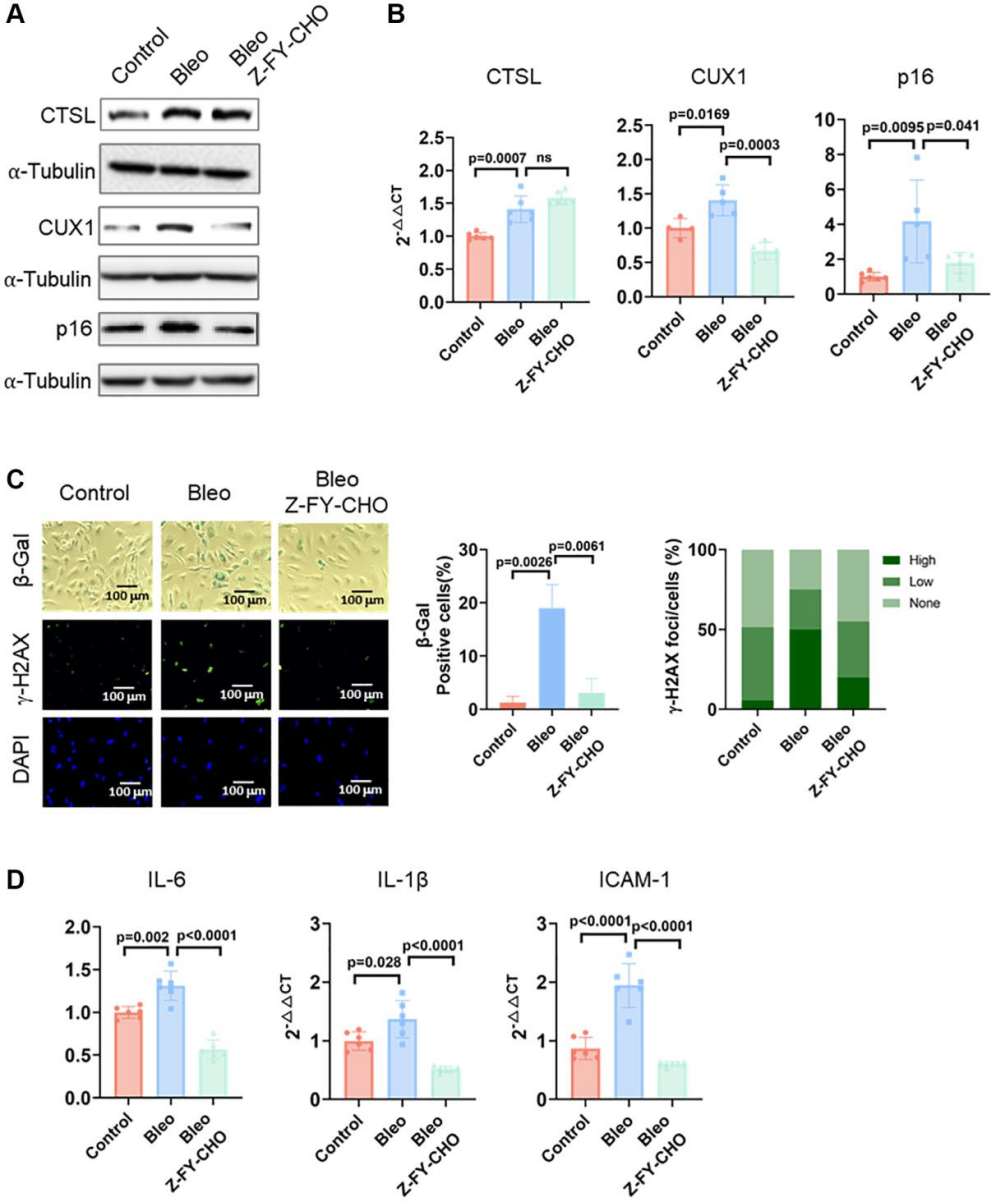
**CTSL is an upstream regulator of CUX1 and p16^INK4a^, inducing DNA damage-induced senescence.** (**A**, **B**) Western blot and qPCR analyses showing that bleomycin (Bleo)-induced activation of CTSL induces CUX1 and p16^INK4a^ expression, which can be reversed by treating the cells with Z-FY-CHO. (**C**, **D**) SA-β-gal and γ-H2AX staining and the expression of the SASP genes *IL6*, *IL-1β*, and *ICAM-1* verify that bleomycin-induced activation of CTSL induces cellular senescence, which can also be reversed by treating the cells with Z-FY-CHO. Quantitative plots for both β-gal^+^ cells (%) in SA-β-gal staining and γ-H2AX foci/cells (%) with γ-H2AX staining were shown on the right side of the panel C. DAPI staining visualizes the presence of nuclei as a control for the cells in this analysis. Data for Western blots represent three biologically independent samples (*n* = 3). Data for qPCR represent a combination of three (*n* = 3) biologically independent experiments. Data for both SA-β-gal staining and γ-H2AX staining represent three biologically independent samples (*n* = 3).

**Figure 6:**
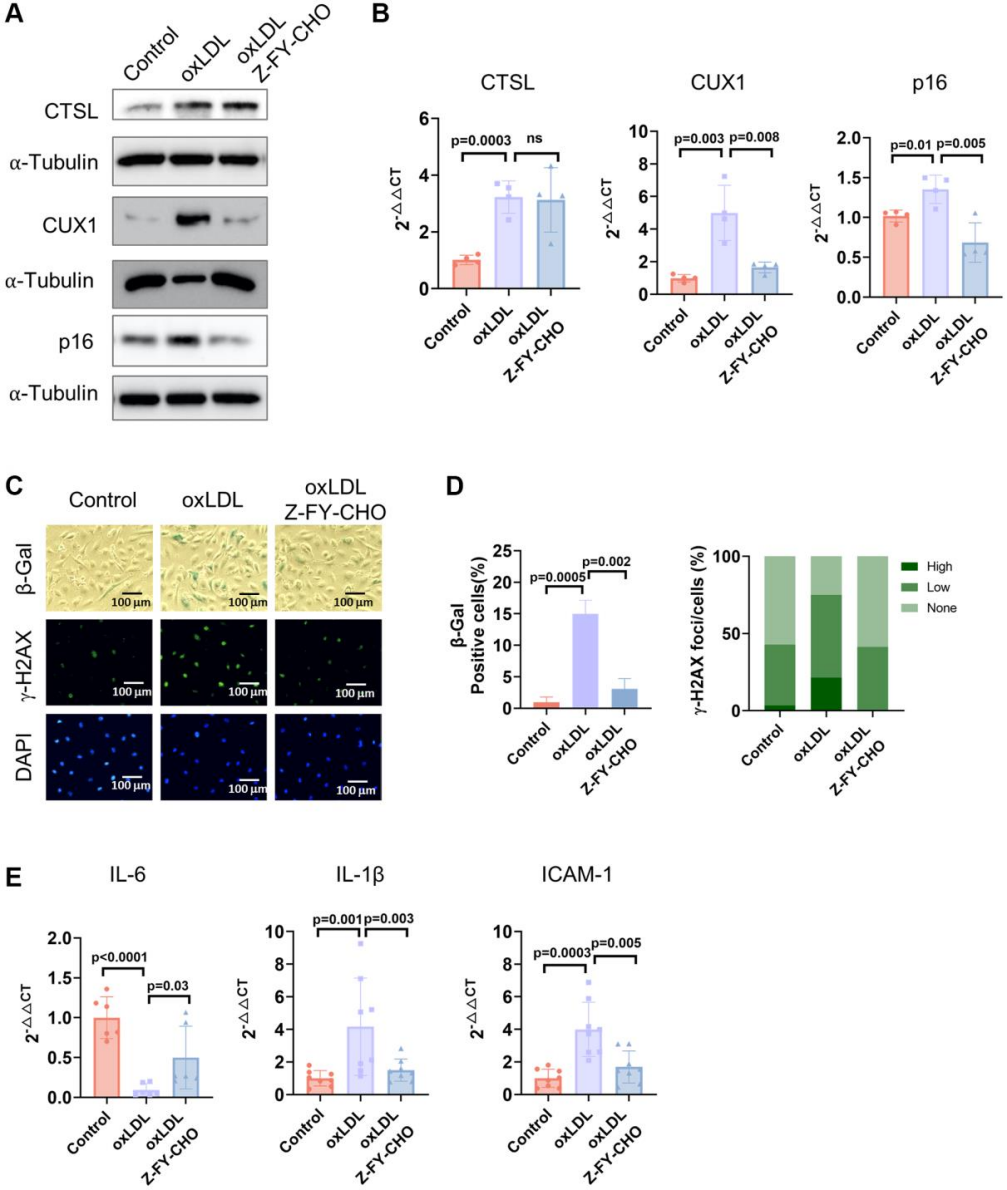
**CTSL is an upstream regulator of CUX1 and p16^INK4a^, inducing oxLDL-induced senescence.** (**A**, **B**) Western blot and qPCR analyses show that oxLDL-induced activation of CTSL induces CUX1 and p16^INK4a^ expression, which can be reversed by treating the cells with Z-FY-CHO. (**C**–**E**) SA-β-gal and γ-H2AX staining and the expression of the SASP genes *IL6*, *IL-1β*, and *ICAM-1* verify that oxLDL-induced activation of CTSL induces cellular senescence, which can also be reversed by treating the cells with Z-FY-CHO. Quantitative plots for both β-gal^+^ cells (%) in SA-β-gal staining and γ-H2AX foci/cells (%) with γ-H2AX staining were shown on the right side of the panel D. DAPI staining visualizes the presence of nuclei as a control for the cells in this analysis. Data for Western blots represent three biologically independent samples (*n* = 3). Data for qPCR represent a combination of three (*n* = 3) biologically independent experiments. Data for both SA-β-gal staining and γ-H2AX staining represent three biologically independent samples (*n* = 3).

Cellular senescence can also be triggered prematurely by various stresses including DNA damage, oxidative stress, oncogenic activation, and metabolic dysregulation [18, 19, 27]. Previously, we demonstrated that both DNA damage and oxidative stress-induced cellular senescence is activated via the CUX1/p16^INK4a^ signal pathway [13]. To determine if CTSL also participates in DNA damage-induced premature senescence, we first checked the expression of CTSL, CUX1, and p16^INK4a^ in human ECs treated with 0.5 μg/ml bleomycin, a genotoxic drug known to induce double-stranded DNA breaks [28]. A significant increase of CTSL, CUX1, and p16^INK4a^ expression was detected at both protein and mRNA levels in the bleomycin-treated human ECs compared to the PBS-treated controls (Figure 5A, 5B, Lane 1 and 2). As expected, an increased expression of CTSL, CUX1, and p16^INK4a^ in the bleomycin-treated human ECs resulted in an induction of cellular senescence as evident by the increased SA-β-gal and γ-H2AX staining (Figure 5C, Column 1 and 2) as well as the upregulated expression of the SASP genes *IL6*, and *IL-1β*, and *ICAM-1* (Figure 5D, Lane 1 and 2), suggesting that CTSL is involved in DNA damage-induced cellular senescence. To further demonstrate that CTSL induces DNA damage-induced senescence via activating CUX1 and p16^INK4a^, we treated the bleomycin-induced human ECs with Z-FY-CHO. We observed a significant restoration of the expression of CUX1 and p16^INK4a^ (Figure 5A, 5B, Lane 2 and 3) as well as of cellular senescence (Figure 5C, Column 2 and 3 and Figure 5D, Lane 2 and 3). In addition, we also applied the same functional complementation assay to investigate oxidative stress-induced cellular senescence by treating the 0.1 mg/ml oxLDL-treated human ECs with 30 μM Z-FY-CHO. Similar results were obtained as shown in Figure 6, demonstrating that CTSL is also involved in oxidative stress-induced cellular senescence. Taken together, these data demonstrate that CTSL is an activator of stress-induced cellular senescence.

## DISCUSSION

**Figure 7:**
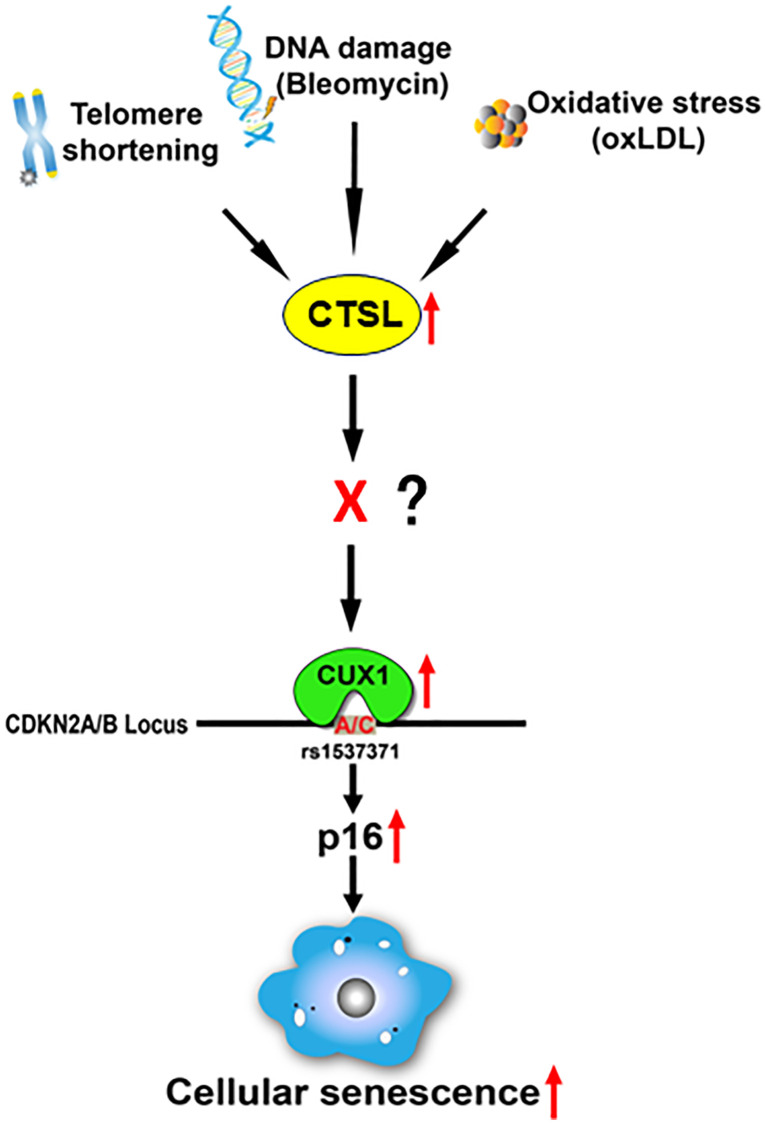
**Proposed diagram showing a signaling pathway that leads to the activation of p16-dependent cellular senescence in response to telomeric shortening, DNA damage, and oxidative stress.** Based on our findings, we believe that telomeric shortening, DNA damage, and oxidative stress can transcriptionally activate CTSL, which results in the transcriptional activation of CUX1. As a result, p16^INK4a^ is activated by CUX1 via its binding to the atherosclerosis-associated fSNP rs1537371, inducing cellular senescence. As CTSL is not a transcription factor and CTSL protease activity is required for the activation of CUX1 as well as cellular senescence, we believe that in between CTSL and CUX1, there is a currently unknown transcription factor X that can be proteolytically activated by CTSL and transcribe CUX1.

Our recent findings showed that CUX1 is an activator of p16^INK4a^, regulating p16^INK4a^-dependent cellular senescence via its binding to an atherosclerosis-associated fSNP rs1537371 on the *CDKN2A/B* locus [13, 29]. In this report, we reveal that CTSL is an upstream activator of CUX1, regulating CUX1 transcription indirectly. Activation of CUX1 by CTSL results in the induction of cellular senescence in response to telomere shortening, bleomycin-induced DNA damage, and oxLDL-induced oxidative stress as shown in Figure 7.

Based on these data, we believe that in between CTSL and CUX1, there might be a yet unknown transcription factor, namely X, as shown in Figure 7. This transcription factor X can be proteolytically activated by CTSL, and its activation can then induce CUX1 transcription. It will be interesting to know what this transcription factor X is and how it is regulated by CTSL. Also, based on our data, we noticed that CTSL is transcriptionally activated in response to telomere shortening, bleomycin-induced DNA damage, and oxLDL-induced oxidative stress as it was detected by qPCR. Thus, it will be equally interesting to know what this transcription factor(s) is, and whether CTSL is activated by the same unknown transcription factor in response to all these senescence triggers or by different transcription factors in response to each of these senescence triggers.

As increasing evidence suggests cellular senescence plays a big role in the pathogenesis of atherosclerosis [30]. Our findings that CTSL activates cellular senescence via an atherosclerosis-associated fSNP on the *CDKN2A/B* locus fully support the senescence theory in the development of atherosclerosis. At the same time, while our data are consistent with the previous publications, indicating that deletion of CTSL in the LDLR^−/−^ mice reduces diet-induced atherosclerosis [8], and increased expression of CTSL in the ECs, VSMCs, and macrophages were observed in human atherosclerotic plaques [31], the same data also provide insights into the signal transduction pathway that leads to atherosclerosis-associated cellular senescence.

Besides CTSL, there are other cathepsins, including cathepsins B, D, X, C, K, and S, that were reported to be involved in atherosclerosis [32–35]. Are all these cathepsins involved in regulating cellular senescence? Cathepsin B was reported localized in nuclei in senescent microglia promoting brain aging [36]; cathepsin K is a potential regulator of osteoclast apoptosis and senescence [37]; and cathepsin S, even though no direct report about its involvement in senescence, its expression is upregulated in the aging and pathological nervous system of mice [38]. Is it possible that different cathepsins can be activated by different signals in different cell types, which induces cellular senescence as well as vascular diseases? Once these questions, among many others, are answered, we will have a deeper understanding about the role of cathepsins in regulating atherosclerosis-associated cellular senescence, which, we believe, will provide us a better strategy to develop precision medicines for atherosclerosis.

In addition, it is believed that both telomeric shortening and DNA damage trigger senescence by inducing genomic instability; they are increasingly recognized as being present in all cells within the atherosclerotic plaque [39, 40]. However, oxLDL was reported to contribute to atherosclerosis by several mechanisms, including the induction of endothelial cell activation, macrophage foam cell formation, and smooth muscle cell migration and proliferation [41]. Our findings that oxLDL induces cellular senescence via an atherosclerosis-associated fSNP on the *CDKN2A/B* locus thus provide another possible mechanism that explains the role oxLDL may play in the pathogenesis of atherosclerosis.

## MATERIALS AND METHODS

### Cell culture and reagents

Primary human arterial ECs (Cat#: CC-2535) and human arterial VSMCs (Cat#: CC-2571) were purchased from Lonza. Cells were passaged by 1: 4 split ratio when they grow to confluence. All cells are free of mycoplasma. ECs were cultured in basal medium EGM-2, and VSMCs in SmGM-2 supplemented with Bullet Kit (Lonza). All cells were cultured at 37ºC in 5% CO_2_ incubator.

### Treatment

To test the effect of stress-induced senescence, ECs were treated with 0.5 μg/ml bleomycin (Cayman, Cat#: 13877) for 24 h and with 0.1 mg/ml oxidized Low Density Lipoprotein (oxLDL) (Invitrogen, Cat#: L34357) for 48 h. ECs were also treated with 30 μM of the CTSL inhibitor Z-FY-CHO (Santa Cruz Biotechnology, Cat#: 167498-29-5) for 48 h.

### qPCR

Total RNA was isolated with the RNeasy Mini kit (Qiagen, Cat#: 74104). cDNA was synthesized with SuperScript^®^ III Reverse Transcriptase (Invitrogen, Cat#: 18080044) after the RNA samples were treated with DNase I (Invitrogen, Cat#: AM8170G). All the procedures were performed following the manufacturer’s protocols. qPCR was performed with the StepOne real-time PCR system according to the protocol for the Power SYBR Green PCR Master Mix (Applied Biosystems, Cat#: A46109) and for TaqMan Universal PCR Master Mix (Applied Biosystems, Cat#: 4318157). The following probe/primer mixes for TaqMan PCR were purchased from Applied Biosystems: CUX1 Hs00738851_m1, p16^INK4a^ Hs02902543_mH, and GAPDH internal control (Hs02786624_g1). Other Primers used, including the primers for SASP genes *IL6* and *IL-1β*, and *ICAM-1*, are listed in Supplementary Table 1. All the data represent the combination of three independent samples (*n* = 3), each with two technical repeats.

### Western blot

Whole cell proteins were isolated with RIPA buffer (Sigma, Cat#: R0278). Proteins were separated on SDS-PAGE gel and transferred to PVDF membranes. Proteins were detected with protein-specific antibodies. All antibodies were purchased and used as listed in Supplementary Table 2. For control, α-tubulin was used. The data represent three independent biological replicates (*n* = 3).

### Senescence β-galactosidase staining

The Senescence β-Galactosidase Staining Kit (Cell Signaling, Cat#: 9860S) was used to detect senescent cells by following the manufacturer’s instruction (https://www.cellsignal.com/products/cellular-assay-kits/senescence-b-galactosidase-staining-kit/9860). Senescent cells were visualized using an RVL-100-G microscope (Echo Laboratories, San Diego, CA, USA). Images were analyzed using ImageJ software (version 1.52K, NIH). The data represent three independent biological replicates (*n* = 3).

### γ-H2AX staining

γ-H2AX staining was performed as previously described [13]. Cells were seeded on glass coverslips on 24 well plates and fixed in 4% paraformaldehyde. Cell membrane was solubilized in PBS containing 5% FBS and 0.5% Triton X-100. Cells were first incubated with γ-H2AX antibodies in the solubilizing buffer for 1 h and then with Alexa Fluor 488-conjugated secondary antibody. γ-H2AX staining was visualized using an RVL-100-G microscope (Echo Laboratories). Images were analyzed using ImageJ software (version 1.52K, NIH). For control, nuclei were stained with 4’, 6-diamidino-2-phenylindole (DAPI) (Sigma, Cat#: D9542). The data represent three independent biological replicates (*n* = 3).

### RNAi knockdown

For siRNA transient CTSL knockdown in human ECs, ON-TARGETplus Human CTSL siRNA was purchased from Horizon Discovery. siRNA transfection was performed by using Lipofectamine RNAiMAX Transfection Reagent (Invitrogen, Cat# 13778150) according to the manufacturer’s protocol (https://www.thermofisher.com/content/dam/LifeTech/migration/en/filelibrary/pdf.par.99253.file.dat/protocols.par.67200.file.dat/huvec-rnaimax.pdf). 24 h after transfection, cells were harvested for RNA isolation.

### Overexpression of CTSL

For overexpression of human CTSL, Cathepsin L expression vector was purchased from Addgene (Cat #: 11250). The full length CTSL cDNA was cloned into pLVX-M-puro vector (Addgene, Cat#: 125839) and confirmed by sequencing. Lentiviruses were generated by transfecting 293T cells with pLVX-CTSL, together with psPAX2 (Addgene, Cat#:12260) and pMD2.G (Addgene, Cat#:12259), according to Addgene’s protocol (https://www.addgene.org/protocols/lentivirus-production/). Generated viruses were infected into human ECs by incubating the virus with 10 μg/ml polybrene for 3 h at 37°C.

### BrdU proliferation assay

Human ECs proliferation was determined by BrdU incorporation using the BrdU Cell Proliferation Assay Kit (Cell Signaling, Cat#: 6813). Briefly, human ECs were subcultured (10,000 cells per well) in 96-well plates for 24 h after virus infection. Then 1× BrdU was added to the culture medium for DNA labeling. The labeling medium was removed after 2 h, then cells were fixed, and DNA was denatured by the addition of 100 μl of fixing/denaturing solution for 30 min. The incorporated BrdU was then detected by a mouse anti-BrdU monoclonal antibody and measured using an anti-mouse IgG, horseradish peroxidase-linked antibody following the manufacturers’ instructions. Data for the BrdU proliferation assay represent *n* = 6 independent biological samples.

### Statistical analysis

*P*-values were calculated using Student’s *T*-test with 2 tails. Error bars represent the median with S.E. Data for Western blots represent three biologically independent samples (*n* = 3). Data for qPCR represent a combination of three (*n* = 3) biologically independent experiments. Data for both SA-β-gal staining and γ-H2AX staining represent three biologically independent samples (*n* = 3). Quantitative plots for both β-gal^+^ cells (%) in SA-β-gal staining and γ-H2AX foci/cells (%) with γ-H2AX staining were shown on the right side of the SA-β-gal staining and γ-H2AX staining panel.

### Data availability

All data and reagents are available upon reasonable request.

## Supplementary Materials

Supplementary Tables
