# Supplementary material for: Cathepsin L induces cellular senescence by upregulating CUX1 and p16INK4a
Source: Aging (Albany NY). 2024 Jun 18;16(13):10749–64. doi: 10.18632/aging.205955 (PMC11272106; doi:10.18632/aging.205955)
Supplement: Supplementary Tables [file aging-16-205955-s001.pdf]

SUPPLEMENTARY TABLES

Supplementary Table 1. Primers used in this paper.

Application	Name	Sequence
qPCR	*CUX1-F*	CCATGGAGTTTGCACCGT
	CUX1-R	CAGCGAGCGGTTCTTCTC
	CTSL-F	GAATCTGGTAGACTGCTCTGGG
	CTSL-R	GATGTCCACAAAGCCGGTGT
	IL6-F	GCAGAAAACAACCTGAACCTT
	IL6-R	ACCTCAAACTCCAAAAGACCA
	ICAM1-F	AGCGGCTGACGTGTGCAGTAAT
	ICAM1-R	TCTGAGACCTCTGGCTTCGTCA
	IL1β-F	ACAGATGAAGTGCTCCTTCCA
	IL1β-R	GTCGGAGATTCGTAGCTGGAT
	GAPDH-F	CGACCACTTTGTCAAGCTCA
	GAPDH-R	AGGGGTCTACATGGCAACTG
shRNA knockdown	*CUX1 shRNA*	GCACGATATTGAAACAGAGAA

Supplementary Table 2. Antibodies used in this paper.

Antibody	Manufacturer	Cat#	Molecular weight	Usage
CTSL	Proteintech	27952-1-AP	29 kDa	WB
CUX1	Abclonal	A2213	200 kDa	WB
α-Tubulin	Thermofisher	T9026	55 kDa	WB
p16	Proteintech	10883-1-AP	16 kDa	WB
γ-H2AX	Santa Cruz	sc-517348	–	IF
Goat anti-Mouse IgG Secondary Antibody, Alexa Fluor™ 488a	Thermofisher	A-11001	–	IF
